# Characteristics, Performance and Microbial Response of Aerobic Granular Sludge for Treating Tetracycline Hypersaline Pharmaceutical Wastewater

**DOI:** 10.3390/microorganisms12061173

**Published:** 2024-06-09

**Authors:** Bichen Lou, Zhonghui Yang, Shengyan Zheng, Dong Ou, Wanpeng Hu, Ning Ai

**Affiliations:** 1 College of Biological, Chemical Science and Engineering, Jiaxing University, Jiaxing 314001, China; loubichen0130@163.com (B.L.);; 2 College of Chemical Engineering, Zhejiang University of Technology, Hangzhou 310014, China

**Keywords:** aerobic granular sludge, antibiotic resistance genes (ARGs), microbial community structure, tetracycline, quantitative polymerase chain reaction

## Abstract

Salt-tolerant aerobic granular sludge(AGS) was successfully cultivated under the dual stress of tetracycline and 2.5% salinity, resulting in an average particle size of 435.0 ± 0.5 and exhibiting a chemical oxygen demand(COD) removal rate exceeding 80%, as well as excellent sedimentation performance. The analysis of metagenomics technology revealed a significant pattern of succession in the development of AGS. The proportion of *Oleiagrimonas*, a type of salt-tolerant bacteria, exhibited a gradual increase and reached 38.07% after 42 days, which indicated that an AGS system based on moderate halophilic bacteria was successfully constructed. The expression levels of targeted genes were found to be reduced across the entire AGS process and formation, as evidenced by qPCR analysis. The presence of *int1* (7.67 log10 gene copies g^−1^ in 0 d sludge sample) enabled microbes to horizontally transfer ARGs genes along the AGS formation under the double pressure of TC and 2.5% salinity. These findings will enhance our understanding of ARG profiles and the development in AGS under tetracycline pressure, providing a foundation for guiding the use of AGS to treat hypersaline pharmaceutical wastewater.

## 1. Introduction

In recent years, the global consumption of antibiotics has continued to rise due to the increasing demand of human health medication and the development of the pharmaceutical industry. The accompanying large-scale discharge of pharmaceutical wastewater has triggered public concern because of its serious pollution to water bodies and potential risks to human health [1]. Pharmaceutical wastewater generally contains recalcitrant organic pollutants, chemical raw material, inorganic salinity, residue drugs, and their derivates [2], which pose substantial challenges to biological wastewater treatment processes. Additionally, the presence of residual antibiotics in aquatic ecosystems pose a significant threat due to their toxic effects and their potential to promote antibiotic-resistant bacteria and antibiotic resistance genes (ARGs) [3,4]. Meanwhile, activated sludge, as the most widely applied biological treatment for pharmaceutical wastewater, may give the concern that the activated sludge environment serves as a fertile platform for antibiotic-resistant bacteria (ARB) propagation and the dissemination of AGRs or as the potential pressure that selects ARBs and facilitates ARG transfer [5]. Antibiotic resistance had emerged as a significant public health threat in the 21st century, and it was projected that antibiotic resistance could result in up to 10 million deaths annually by 2050 [6,7]. Thus, given the increase in the variety and concentration of antibiotics and the potential risk of ARGs to human health [4], it is particularly important to study their influence on emerging wastewater treatment processes.

Aerobic granular sludge technology (AGS) has been hailed as the next generation of sewage treatment technology [8,9,10], effectively addressing the drawbacks associated with traditional reactors such as large floor space requirements, high investment costs and energy consumption [11,12,13]. As an advanced technology in the field of sewage biological treatment, AGS had gained increasing attention from researchers due to its outstanding characteristics, including high sludge concentration, excellent settling performance, compact structure, impact resistance, and anti-toxicity [14,15,16]. In addition, the unique three-dimensional structure of AGS provides effective protection for internal microorganisms from external influences due to mass transfer resistance, which creates aerobic, anoxic and anaerobic zones within the granules, thereby enhancing system stability and removal efficiency in AGS [17,18]. Pharmaceutical wastewater contains refractory organic matter and high levels of inorganic salinity; the concentration ranges of inorganic salts in pharmaceutical wastewater are 13–15,000 mg L^−1^, and the COD concentration ranges from 262–98,000 mg L^−1^ based on 229 literature studies [19]. Chen et al. [4] evaluated the performance of the AGS system in municipal wastewater containing mixed antibiotics; the results demonstrated that the average removal efficiencies of four antibiotics were 79.17% (TC), 70.86% (SMX), 25.73% (OFL), and 88.93% (ROX), respectively. However, because of its unique three-dimensional spatial structure, AGS could protect the internal bacteria from the adverse external environment to a certain extent [17,20,21]. Moreover, the introduction of salinity did not exert any adverse impact on granulation with non-adapted sludge [22]. A salinity level of up to 7.5 g·L^−1^ was found to promote biomass growth [22]. Thus, it was necessary to evaluate the characteristics, long-term performance and microbial response of AGS for the treatment of hypersaline pharmaceutical wastewater.

Additionally, wastewater treatment plants (WWTPs) were recognized as a significant reservoir of antibiotics, ARGs and antibiotic-resistant bacteria (ARB), which could be released into the environment [23]. The removal of antibiotics and ARGs varies in biological treatment [24]. Some studies found that WWTP treatment increased the concentration of antibiotics and some ARGs after WWTP treatment [25]. Other studies found that activated sludge based on biological treatment decreased the relative concentration of antibiotics and ARGs in WWTP [24,25,26]. As a relatively new treatment technology, the effects of AGS on antibiotic concentration and the relative abundance of ARGs in the treatment of pharmaceutical wastewater are still unclear. Considering the fact that certain antibiotics may also promote the selection of ARGs related to other types of antibiotics in wastewater treatment plants [23,27], further efforts are required to evaluate the impact of antibiotics on the development of non-corresponding ARGs in the AGS system.

In this paper, a lab-scale SBR system was set up investigate the long-term impact of tetracycline hypersaline pharmaceutical wastewater on the formation and performance of AGS. Based on this premise, the dynamic shifts in microbial communities within AGS under typical antibiotic pressure were analyzed, and the interplay between the structure and function of granular sludge community, as well as the linkage effect of key microorganisms/genes and major resistance genes was explored. In addition, the dynamic changes of ARGs, including *tetA*, *tetW*, *sulI*, *sulII*, and *bla_TEM-1_*, corresponding to tetracycline as well as other types of antibiotics in AGS, were investigated. Additionally, the impact of tetracycline on the horizontal gene transfer of ARGs in AGS was evaluated by determining the presence of class I integrase (*intI1*). The research results not only have important academic value, but also provide a theoretical basis for the engineering application of AGS technology in the treatment of hypersaline pharmaceutical wastewater.

## 2. Materials and Methods

### 2.1. Reactor Start-Up and Operation

For the cultivation of AGS, a laboratory-scale sequencing batch reactor (SBR) was constructed with a working volume of 1 L (40 cm in height and 6 cm in diameter). The entire reactor system was managed through various time controllers to achieve automated operation. Generally, the reactor operated through five distinct processes in each cycle: feeding, anoxic phase, aerobic phase, settling, and discharge. In order to enhance the efficiency of the reactor and facilitate the formation of AGS, various operational parameters were adjusted and optimized over a period of 42 days [28]. The detailed operating parameters are summarized in Table 1. The volume exchange ratio (VER) of the reactor was set to 50%, and the aeration rate remained constant at 3 L/min. The operating temperature was maintained at 22 ± 2 °C.

### 2.2. Seed Sludge and Influent Composition

The seed sludge utilized in this study was obtained from the recovered sludge of the secondary clarifier at the Jiaxing municipal wastewater treatment plant in China. The seed sludge exhibited a mixed liquor suspended solids (MLSS) concentration of 22.134 g/L and a sludge volume index (SVI_5_) of 28.463 mL/g.

The composition of the synthetic hypersaline pharmaceutical wastewater, including the presence of TC, salinity, and various chemical constituents at specific concentrations is summarized in Table 2. The glucose and TC were served as the carbon source, and the influent organic concentration remained 1000 mg COD L^−1^, corresponding to an organic loading rate (OLR) of 2 kg COD m^−3^ d^−1^. The influent pH was adjusted to 7.0 ± 0.1 by adding NaHCO_3_ or HCl. 2.5 mL trace elements to the influent for microbial growth, and its composition is summarized in Table 3 [29,30].

### 2.3. Conventional Analysis Methods

The sludge volume index (SVI_5_ and SVI_30_), mixed liquor suspended solids (MLSS), mixed liquor volatile suspended solid (MLVSS), and chemical oxygen demand (COD) were determined using the standard method [31,32]. Mercury sulfate was employed to mitigate the interference of chloride in COD measurement [33]. The particle size of the granular sludge was determined by a laser particle size analyzer (Malvern Mastersizer, Singapore). The specific oxygen uptake rate (SOUR) was used to assess the microbial activity in the AGS system. The procedure is outlined as follows [34]: After aeration and agitation, DO was maintained in the range of 4–6 mg/L, then the rate of the oxygen uptake rates (OUR) was measured after the raw sludge was washed three times. The DO was collected by using a high-precision sensor (YSI DO200A, Yellow Springs, OH, USA). To eliminate the effect of the MLSS on OUR, the specific oxygen uptake rate (SOUR) was calculated based on the following formula:

SOUR(mgO_2_(gMLSS)^−1^ h^−1^) = OUR/MLSS (1)

### 2.4. Metagenomic Sequence, Assembly and Analysis

Samples of granular sludge collected at 0 d, 12 d, 24 d, and 48 d were selected as study samples and labeled as d0, d12, d24, and d48, respectively. Microbial genomic DNA was extracted from samples using the OMEGA Soil DNA Kit (D5625-01) according to the manufacturer’s instructions and stored at −20 °C for further analysis. The extracted DNA was analyzed using agarose gel electrophoresis and a Nano Drop spectrophotometer (ND-1000 Fisher Scientific, Waltham, MA, USA) for detection and evaluation. The extracted microbial DNA was processed to construct metagenome shotgun sequencing libraries with insert sizes of 400 bp by using the Illumina TruSeq Nano DNA LT Library Preparation Kit. Each library was then sequenced on a Novaseq 6000 platform (Illumina, San Diego, CA, USA) with the PE150 strategy at Personal Biotechnology Co., Ltd. (Shanghai, China). The sequencing data underwent rigorous quality evaluation and control, with contamination being eliminated upon meeting the required standards. Subsequently, high-quality datasets suitable for downstream metagenomic analysis were obtained and subjected to bioinformatics analysis. Further detailed information of the analysis process can be found in the Supplementary Material.

All targeted metagenomics sequencing data were subsequently searched for antibiotic resistance genes (ARGs) against the Comprehensive Antibiotic Resistance Database (CARD) v3.0.5, which includes 478 specific ARGs. For annotation purposes, the CARD database v3.0.5 was downloaded and DIAMOND v0.7.0.49 was utilized to annotate the sequences, with only the hits having a sequence identity >95% being retained [35]. The annotation output was then converted into TPM values representing the relative gene abundance in each sludge sample.

### 2.5. Quantitative PCR of Selected ARGs

To assess the performance of AGS in terms of ARGs removal, as well as to investigate its occurrence throughout the granulation process and track its transfer dynamics, a molecular analysis utilizing the quantitative polymerase chain reaction (qPCR) was conducted to detect a selected panel of genes from three biological replicates of samples listed in Table 4. The selected AGRs were derived from the antibiotic classes of tetracyclines (tetA, tetW), sulfonamides (*sul1*, *sul2*), and beta-lactams (*bla_TEM_*). These ARGs were chosen based on antibiotics consumption and how often they are detected in the water environment [26,30,36,37]. The 16s rRNAs were quantified for the normalization of the gene copies to the concentration of bacteria in sludge samples. Meanwhile, the int1 was employed to evaluate the ARGs’ mobility, acquisition, and exchange between microbes [38].

The removal ability of the ARGs was calculated by comparing the concentration of target genes between 0 d and 42 d sludge samples. The calculation of the ARGs was used as shown below:

Log removal = Log10_ARGs_(0 d AGS) − Log10_ARGs_(42 d AGS)

### 2.6. Statistical Analysis

The results of the qPCR of the selected ARGs were presented as the arithmetic mean with the corresponding standard deviation (n = 3). The statistical significance of the differences observed among treatments was determined via the one-way analysis of variance (ANOVA) and covariance (ANCOVA), followed by Tukey’s pair-wise comparison at a significance level of *p* < 0.05.

## 3. Results and Discussion

### 3.1. Structure Characteristics of AGS

**Figure 1:**
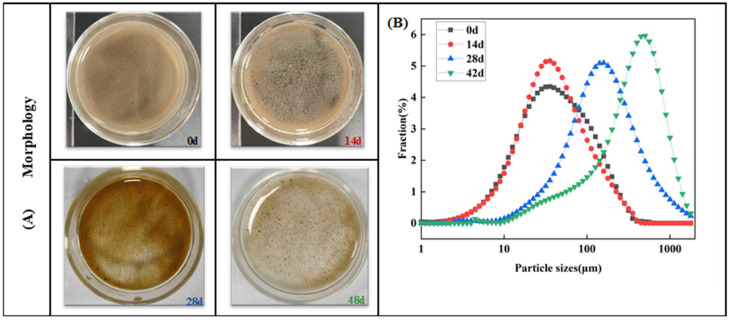
Morphology and particle size distribution of sludge during aerobic granulation: (**A**) Morphology of granular sludge and (**B**) particle size distribution.

The formation process of AGS was monitored on both macro and micro scales. From inoculation to day 14 (Figure 1A), the activated sludge exhibited a loose, flocculent state with a mean diameter of only 60.5 ± 1.8 μm. The small flocs with a mean diameter of 56.5 ± 0.2 μm were observed after continuous cultivation for 14 days. After 28 days of cultivation, some visible micro-sludge particles were detected (Figure 1A), and the mean diameter correspondingly increased from 56.5 ± 0.2 μm to 229.7 ± 2.6 μm (Figure 1B). The stable sludge particles were primarily formed after 42 days of incubation (Figure 1B), exhibiting a mean diameter of 435.0 ± 0.5 μm, which was approximately six times larger than the mean diameter of the sludge at 0 d. The results demonstrated that AGS was successfully cultivated with a particle size of 435.0 ± 0.5 μm under the double pressure of antibiotics and salinity (2.5%) after 48 days.

The structural characteristics of AGS (42 d) were observed via SEM, revealing an ellipsoidal shape with a distinct boundary and compact structure in the salt-tolerant aerobic granular sludge. Upon further magnification (Figure S1), uneven particle surfaces with pores and gaps were also observed. These pores and gaps could serve as conduits for the transfer of the nutrient matrix and oxygen into the interiors of the particles, while simultaneously allowing for the discharge of microorganism metabolites from said pores and gaps. When oxygen diffused into the particle, it created aerobic, facultative, and anaerobic zones due to mass transfer resistance. These zones significantly enhanced the microbial diversity within the granule and promoted the efficient removal of refractory organic matter by aerobic granular sludge [17].

### 3.2. Performance of AGS

#### 3.2.1. Carbon Removal Efficiency

**Figure 2:**
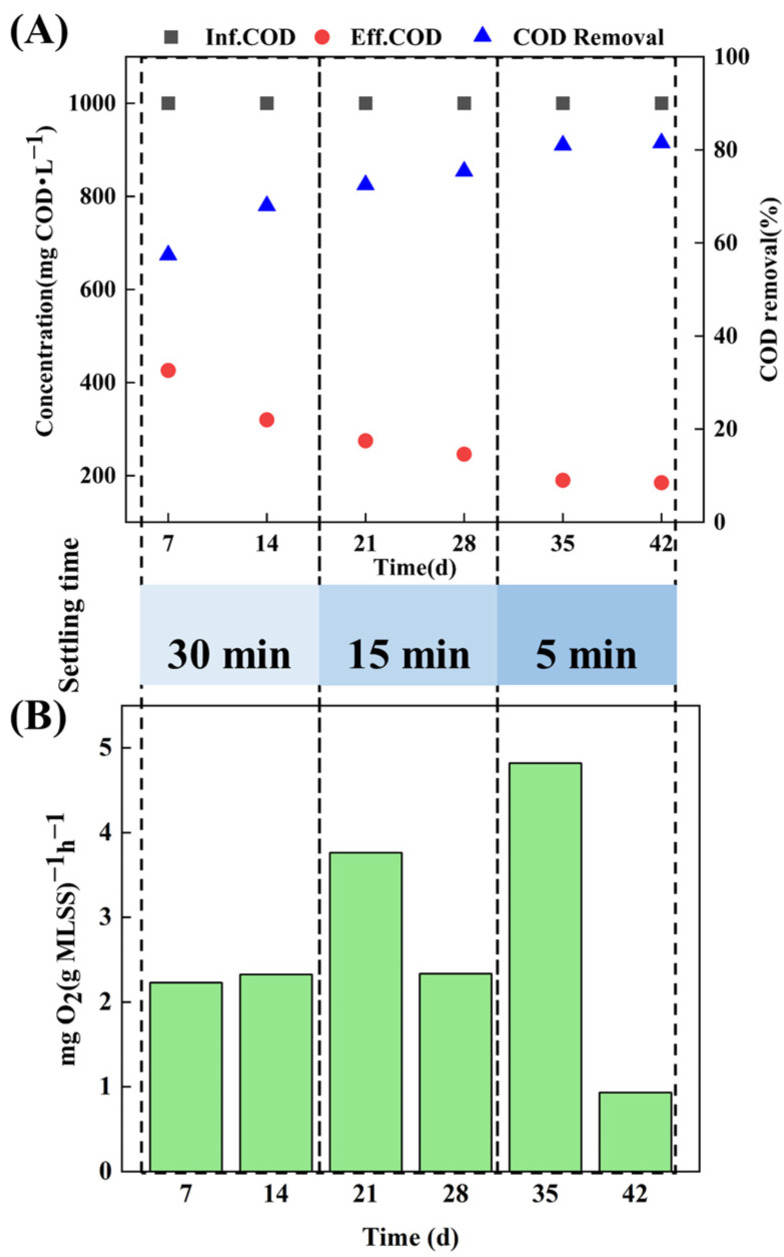
Removal performance during aerobic granulation: (**A**) COD removal rate and (**B**) SOUR.

The carbon removal efficiency of the AGS system was characterized by the COD removal rate and SOUR in treating target wastewater (Figure 2). The granulation process resulted in an improvement of the COD removal efficiency in the AGS system. In detail, in the stage of 0–14 d, with operating conditions of 2.5% salinity and a settling time of 30 mins, the COD removal rate of the sludge system was maintained between 57.4% and 68%, and SOUR remained at 2.3 mgO_2_(g MLSS)^−1^ h^−1^, which further confirmed the fact that in the early stage of particle formation, the system was greatly affected by salinity and tetracycline, and the microbial activity was inhibited. In the stage of 14–28 d, as AGS gradually formed, the COD removal rate increased to 75.4%, while SOUR in the SBR system decreased from 3.763 mgO_2_(g MLSS)^−1^ h^−1^ to 2.335 mgO_2_(g MLSS)^−1^ h^−1^. In the subsequent stage of 28–42 d, as AGS matured further, a stable COD removal rate above 80% was achieved for treating tetracycline hypersaline pharmaceutical wastewater.

#### 3.2.2. Sludge Concentration

**Figure 3:**
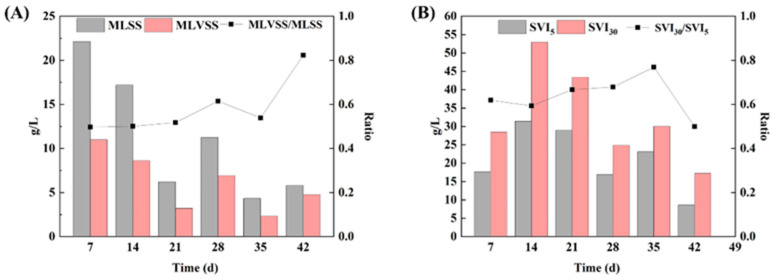
Sludge settlement performance during aerobic granulation: (**A**) MLSS, MLVSS, and MLVSS/MLSS; and (**B**): SVI_30_, SVI_5_, and SVI_30_/SVI_5_.

The sludge concentration during AGS granulation exhibited a pattern of initial decline followed by a subsequent increase (Figure 3A). The MLSS decreased from 22.314 g/L to 17.190 g/L within the first 14 days (Table 5). In the stage of 14–21 d, the settling time was shortened from 30 min to 15 min due to the selective retention of sludge with superior settling performance and salt-tolerance ability in the reactor, resulting in a decrease in the MLSS to 6.219 g/L. In the stage of 21–28 d, granules with regular morphology were observed, leading to the further enhancement of the salt resistance and sedimentation performance of AGS, and the MLSS increased to 11.255 g/L. However, in the stage of 28–35 d, the settling time was reduced to 5 min and, with the further enhancement of hydraulic selective pressure, the MLSS decreased to 4.332 g/L. After a cultivation period of 7 days, the MLSS recovered to 5.795 g/L. These results demonstrated that, with the gradual maturity of AGS, the sludge concentration in the reactor increased, and more sludge with salt resistance and good settling performance remained in the reactor.

#### 3.2.3. Settlement Performance

The profiles of sludge settlement performance throughout the entire cultivation process of AGS are illustrated in Figure 3. The SVI remained consistently below 55 mL/g throughout the entire 42-day cultivation period. Additionally, the SVI_30_/SVI_5_ exhibited an increase from 0 d to 35 d, leading to an improvement in granule settling properties. In the stage of 0–14 d, there was a gradual increase in SVI_30_ from 17.620 mL/g to 31.414 mL/g, indicating that the inoculated sludge was gradually adapting well to the high salt system, and the settlement performance was decreased. In the stage of 14–28 d (settling time was 15 min), SVI_30_ decreased from 28.944 mL/g to 16.881 mL/g, while in the subsequent stage of 28–42 d (settling time was 5 min), SVI_30_ further decreased from 23.084 mL/g to 8.628 mL/g. Good settleability is one of the main characteristics of AGS, as well as the guarantee of short settling time without being washed out by SBR system [17,21]. This highlights the application prospect of AGS technology in treating tetracycline hypersaline pharmaceutical wastewater.

### 3.3. Microbial Population Dynamics of AGS

#### 3.3.1. Characteristics of Microbial Community

The sludge samples from 0 d, 14 d, 28 d, and 42 d were collected to characterize the succession of the microbial communities in AGS for treating tetracycline hypersaline pharmaceutical wastewater. All samples were collected under identical operating conditions to minimize operational errors [17]. The rarefaction curve (Figure S2A) was utilized to compare the species among four sludge sample sequences and assess the representativeness of the collected sequences [17]. The results demonstrated that sample d0 exhibited the highest species richness, which decreased significantly with increasing culture time (Table 6). This finding is consistent with our previous research and may be attributed to the selection pressure of high salinity and tetracycline. The inadaptability of certain microorganisms to this environment led to their gradual elimination from the system, resulting in a decline in community richness [17]. The Shannon diversity index and the Simpson diversity index were employed to assess the community diversity [39]. The findings indicated that sample d0 exhibited the highest species richness and diversity. As tetracycline pressure and 2.5% salinity increased, the species diversity of the aerobic granular sludge in the SBR reactor gradually decreased with prolonged incubation time. In addition, the Venn diagram (Figure S2B) showed that there were a total of 18,680 species present in the sludge samples collected at four different incubation times. However, under tetracycline pressure and 2.5% salinity pressure, the unique species in the granular sludge gradually decreased from 1996 on d0 to 473 on d42. These results indicated that the microbial community underwent significant changes in treating tetracycline hypersaline pharmaceutical wastewater. Some microorganisms adapted to the dual stress, thrived, and dominated in the SBR system, while others that were unable to adapt gradually disappeared. Meanwhile, the number of species shared between the adjacent sludge samples d0 and d14, d14 and d28, and d28 and d42 were 22,600, 20,437, and 19,617 respectively, which indicated that there were many similarities in the microbial community structure of adjacent sludge samples cultured over a 14-day interval. Furthermore, it indicated that microbial community succession was a gradual process in the formation process of AGS.

#### 3.3.2. Dynamic Succession of Microbial Community

**Figure 4:**
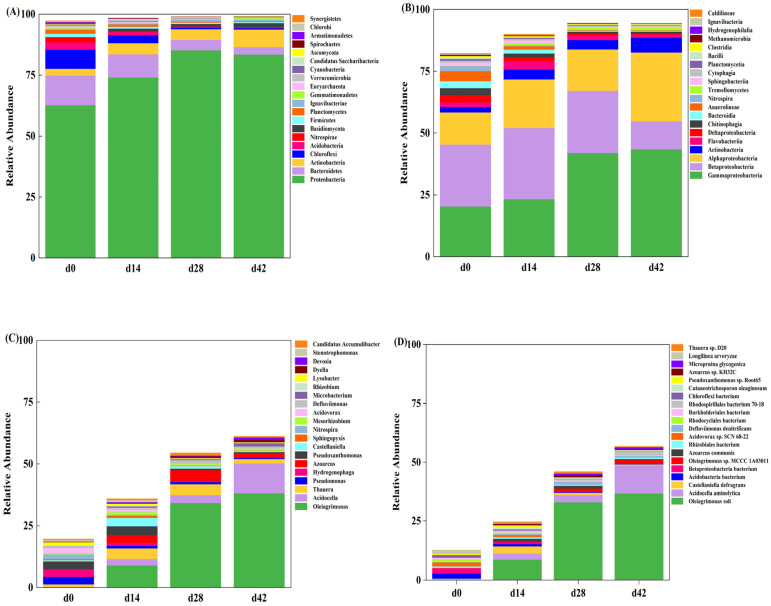
Analysis of the microbial diversity at different taxonomic level for sludge samples: phylum (**A**), class (**B**), genus (**C**), and species (**D**).

The dynamic succession of the microbial community of AGS was monitored at the four taxonomic levels of phylum, class, genus, and species. As shown in Figure 4A, *Proteobacteria* dominated the microbial community of granular sludge at the gate level, with relative abundances of 62.64% (d0), 74.04% (d14), 85.23% (d28), and 83.45% (d42), respectively. The microbial community structure was similar to that of other environmental samples, such as soil [40] and sewage [41].The second dominant phylum was *Bacteroidetes*, accounting for 12.18% (d0), 9.51% (d14), 4.24% (d28), and 3.06% (d42) of the microbial community, respectively. *Proteobacteria* is known to thrive in adverse environmental conditions [17,42,43] and has been detected in all water samples containing antibiotics, dominating the microbial community structure with a high relative abundance [42]. Additionally, *Proteobacteria* was identified as the dominant phylum in saline wastewater biological treatment systems, with its relative abundance increasing from 33.4% to 79.6% and 85.1% as salt concentration increased from 0 g·L^−1^ to 50 g·L^−1^ [43].

Figure 4C clearly illustrates the dynamic alterations of the microbial community structure throughout the entire AGS process, furnishing more intricate insights and profound comprehension regarding the succession of microbial communities. Under the stress of tetracycline pressure and 2.5% salinity pressure, the microbial community structure of AGS was significantly different at the genus level, and some genera showed a continuous increase or decrease trend with the extension of culture time. Specifically, the relative abundances of *Pseudomonas* and *Hydrogenophaga* were higher in d0 sludge sample and were dominant in the SBR system. However, with the extension of the culture time, the relative abundances of *Pseudomonas* and *Hydrogenophaga* were only 0.49% and 0.12% in the d42 sludge sample. On the contrary, the relative abundance of *Oleiagrimonas* was only 0.14% at 0 days, but with the extension of culture time, the relative abundance of *Oleiagrimonas* gradually increased to 8.98%, 34.21%, and 38.07% (d14, d28, and d42), becoming the highest abundance genus in the AGS system. This increasing dynamic change demonstrated that *Oleiagrimonas* could adapt to the double stress of tetracycline pressure and 2.5% salinity pressure, had a great contribution to the overall salt tolerance of the granular sludge system, and was the dominant and functional genus in the AGS system.

From the point of view of the dominant microbe and the functional microbe, the existence and enrichment of *Oleiagrimonas* were the keys to the survival and growth of AGS under tetracycline pressure and 2.5% salinity pressure. At the species level analysis (Figure 4D), the results showed that the dominant microbes in the d42 sludge sample were composed of *Oleiagrimonas* soli (36.62%) and *Oleiagrimonas* sp. *MCCC 1A03011* (1.45%). *Oleiagrimonas* soli, isolated from the saline oilfield in Shandong Province (China), was a halophilic strain with a good aromatic hydrocarbon degradation ability [44]. The optimum temperature for the growth of *Oleiagrimonas* soli was 20–30 °C, the pH range was 4–10 (the optimum pH was 6–8), and the NaCl range in in the nutrient solution of *Oleiagrimonas* soli was 0.5–10% (*w*/*v*) (the optimum NaCl range was 1.5–5%, *w*/*v*) [44].

### 3.4. Occurrence and Distribution of ARGs in AGS

**Figure 5:**
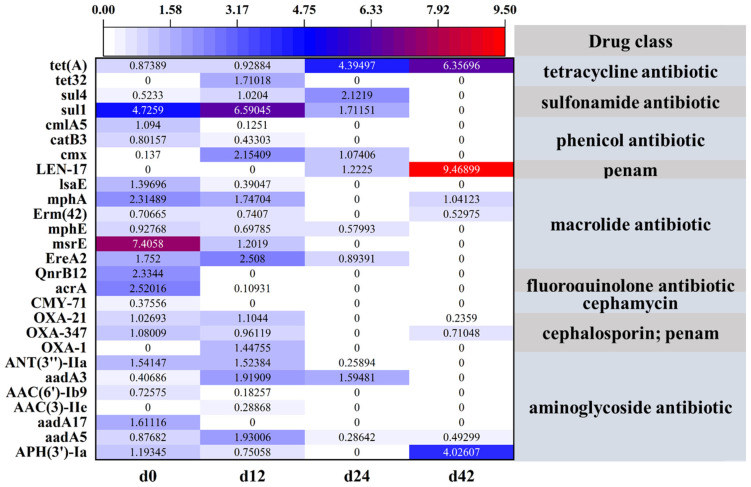
Annotation of the CARD database of four sludge sample under the long pressure of TC.

Through the annotation of the CARD database [35], a total of 160 ARGs were identified, including over 20 drug class from 4 sludge samples (Table S1). To further analyze the variation in ARGs in relation to the formation of specific antibiotic groups, only hits with sequence identities greater than 99.5% were retained. As shown in Figure 5, a total of 27 ARG subtypes, including 9 drug classes, were identified across 4 sludge samples. In detail, *msrE* associated with macrolide resistance and *sul1* associated with sulfonamide resistance were the dominant ARGs in the d0 sludge samples, and the relative abundances were 7.4058 and 4.7259, respectively. After 14 d cultivation, the ARG for sulfonamide (*sul1*) exhibited the highest relative abundance compared with the other ARGs in the sludge sample. In the d28 sludge sample, only 10 ARGs were detected based on the CARD database, and *tet*(*A*) associated with tetracycline resistance was dominant in the AGS system. In the d42 sludge sample, only eight ARGs were identified, with *tet*(*A*), *LEN-17*, and *APH*(*3*)*-Ia* being the dominant ones associated with tetracycline, penam, and aminoglycoside resistance, respectively. It is worth noting that the relative abundance of *tet*(*A*) associated with tetracycline resistance stepwise increased under the long pressure of TC and 2.5% salinity.

**Figure 6:**
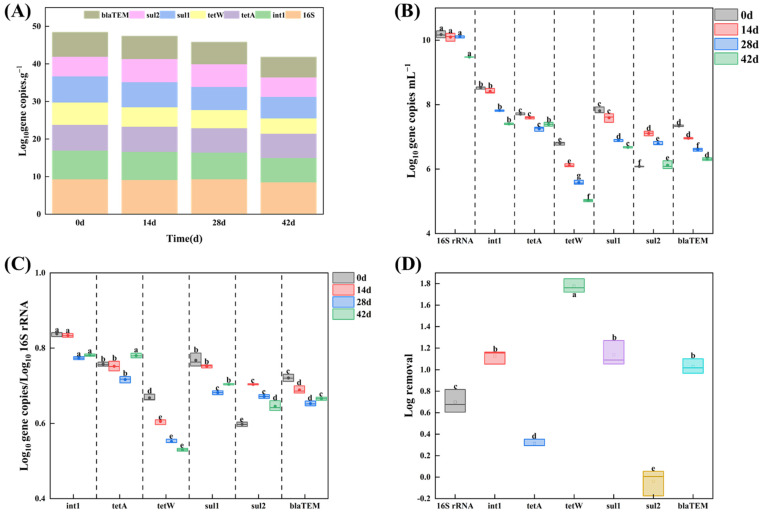
(**A**) Concentration of selected ARGs and *int 1* for SAGS under long pressure of TC. Error bars represent the standard deviation between biological replicates (n = 3). (**B**) Absolute abundance of ARGs and *int 1* expressed in log10 gene copies μL^−1^. (**C**) Relative abundance of genes expressed as log10 gene copies/log10 16S rRNA copies. (**D**) Log removal of AGRs. Values with superscript letters a, b, c, d, e and f are significantly different across columns (*p* < 0.05).

To further quantify the ARGs’ frequency of occurrence and the related removal ability of AGS under the long pressure of TC and 2.5% salinity, six selected ARGs and intI1 as MGE were analyzed by qPCR. The expression levels of the targeted genes were found to be reduced across the entire the AGS process and formation, as evidenced by qPCR analysis. In detail, the targeted genes concentration dropped from 48.44 log10 gene copies g^−1^ to 41.84 log10 gene copies g^−1^ (Figure 6A). This result is in agreement with recent studies [26,45]; that is, AGS technology has a certain reduction in ARGs. Additionally, for the 0 d sludge sample (seed sludge), all selected ARGs were detected, which endowed the microorganisms in the sludge with the capacity to withstand multiple antibiotics, such as tetracycline, sulfonamide, penicillin, and cefradine. The presence of int1(7.67 log10 gene copies g^−1^ in 0 d sludge sample) enabled the microbes to horizontally transfer ARGs genes along the AGS formation under the double pressure of TC and 2.5% salinity. As shown in Figure 6B, a significant decrease in the majority of the targeted genes was observed with the AGS process. *int 1* was the most abundant gene in all targeted genes in the 0 d sludge sample (8.5 log10 gene copies mL^−1^). After 42 d of cultivation, *int 1* and *tetA* were the dominant AGRs in the AGS system; the absolute abundance was 7.4 and 7.39 log10 gene copies mL^−1^ respectively. The other selected ARGs, including *tetW*, *sul1*, and *bla_TEM_*, were all deceased in different degrees (Figure 6D). In detail, the ARGs with log removal > 1 were *int 1* (1.1 log), *tetW* (1.8 log), *sul1* (1.1 log), and *bla_TEM_* (1.0 log), respectively, while those with log removal < 1 were 16S rRNA (0.7 log), *tetA* (0.9 log), and *sul2* (−0.1 log), respectively. Sabri et al. [26] found that the application of AGS technology as an additional treatment resulted in a significant reduction in ARGs, with log removal ranging from 0.08 log (*ermB*) to 2.02 (*tetW*), indicating its effectiveness in mitigating ARGs. Based on the remaining ARGs in the AGS system at 42 d, a total of 40.3 log10 gene copies g^−1^, excluding 16s rRNA, were still present.

## 4. Conclusions

In this study, AGS was successfully cultivated for treating tetracycline hypersaline pharmaceutical wastewater and had good removal performance and sedimentation performance. During the AGS formation, the relative abundance of *Oleiagrimonas* increased gradually and reached 38.07% on day 42, resulting in the successful construction of AGS with moderate salt tolerance. The qPCR results indicated that the AGS technology could partially reduce the ARGs, and the presence of int1 facilitated the horizontal transfer of ARGs among the microbes during AGS formation under dual pressure from TC and 2.5% salinity. The present study will improve our understanding of ARG profiles and the development of AGS under tetracycline pressure, laying the groundwork for using AGS to treat antibiotic-containing wastewater more effectively.

## Figures and Tables

**Table 1:** Operational parameters of the SBR reactor.

Running Time (d)	Cycle Time (h)	Salinity (%)	Feeding (min/Cycle)	Anoxic Time (min/Cycle)	Aerobic Time (min/Cycle)	Settling Time (min/Cycle)	Discharge (min/Cycle)
0–7	6	2.5	2	30	296	30	2
7–14	6	2.5	2	30	296	30	2
14–21	6	2.5	2	30	311	15	2
21–28	6	2.5	2	30	311	15	2
28–35	6	2.5	2	30	321	5	2
35–42	6	2.5	2	30	321	5	2

**Table 2:** Synthetic hypersaline pharmaceutical wastewater constituents.

Constituent	Concentration (mg/L)
Tetracycline (TC)	0.5
Salinity (NaCl)	25
C_6_H_12_O_6_	937
NH_4_Cl	500
KH_2_PO_4_	140
CaCl_2_	150
MgCl_2_	31
FeSO_4_·H_2_O	10

**Table 3:** Trace Element Solution Composition.

Trace Element	Concentration (mg/L)
H_3_Bo_3_	50
ZnCl_2_	50
CuCl_2_	30
MnSO_4_·H_2_O	50
(NH_4_)_6_Mo_7_O_24_·H_2_O	50
AICl_3_	50
CoCl_2_·6H_2_O	50
NiCl_2_	50

**Table 4:** ARGs and MGE selected for qPCR analysis with corresponding descriptions.

Category	Gene	Description
Bacteria normalization	16S rRNA	RNA component of the 30S small subunit of prokaryotic ribosome
ARGs	tetA	Tetracycline resistance protein tetA
	tetW	Tetracycline resistance protein tetW
	sul1	Sulfonamide resistant dihydropteroate synthase
	sul2	Sulfonamide resistant dihydropteroate synthase
	blaTEM	Bla-cefotaxine-hydrolizing β-lactamase
MGE	Int1	Class 1 integron integrase

**Table 5:** Performance of aerobic granular sludge at different settling periods.

Settling Time (min/Cycle)	Salinity (%)	Time (d)	MLSS (g/L)	MLVSS (g/L)	VSS/SS	SVI_5_ (mL/g)	SVI_30_ (mL/g)	SVI_30_/SVI_5_
30	2.5	00–07	22.134	11.964	0.495	28.463	17.620	0.619
30	2.5	07–14	17.190	6.581	0.500	52.938	31.414	0.593
15	2.5	14–21	6.219	3.220	0.518	43.415	28.944	0.667
15	2.5	21–28	11.255	6.917	0.615	24.878	16.881	0.679
5	2.5	28–35	4.332	2.335	0.539	30.009	23.084	0.769
5	2.5	35–42	5.795	4.772	0.823	17.256	8.628	0.500

**Table 6:** Diversity analysis of species for different sludge samples.

Sample ID	Reads	Q30(%)	Community Richness		Community Diversity	
			ACE ^a^	Chao1 ^a^	Shannon ^b^	Simpson ^b^
d0	62919090	94.85	26755	26971	10.43	0.9961367
d14	62348856	94.93	25493	25638	9.96	0.9890374
d28	56729842	94.61	22994	23094	7.85	0.8892812
d42	63234358	95.03	22980	23237	6.81	0.850424

The indices of Chao, ACE, Shannon, and Simpson were calculated at the same sequence depth for four sludge samples. ^a^ Community richness. A higher number represented more richness. ^b^ Community diversity. A higher number represented more diversity.

## Data Availability

All raw sequences were deposited in the NCBI Sequence Read Archive under accession number SRR18495454, SRR18495455, SRR18495453 and SRR18495452 accessed on 1 June 2023.

## Supplementary Materials

The following supporting information can be downloaded at: https://www.mdpi.com/article/10.3390/microorganisms12061173/s1, Materials and methods: Metagenome DNA Extraction and Shotgun Sequencing and Metagenomics Analysis; Figure S1: Microstructure of salt-tolerant aerobic granular sludge by SEM; Figure S2: (A) Rarefaction curve and (B) Venn diagram for sludge samples; Table S1: Primer sequences and PCR conditions used for qPCR amplification in the study and their respective amplification sizes; Table S2: Annotation of CARD database for SAGS.
