# Supplementary material for: Characteristics, Performance and Microbial Response of Aerobic Granular Sludge for Treating Tetracycline Hypersaline Pharmaceutical Wastewater
Source: Microorganisms. 2024 Jun 9;12(6):1173. doi: 10.3390/microorganisms12061173 (PMC11206034; doi:10.3390/microorganisms12061173)
Supplement: Supplementary file 1 [file microorganisms-12-01173-s001.zip › microorganisms-3039378-supplementary.pdf]

Supplementary material

Metagenome DNA Extraction and Shotgun Sequencing

Total microbial genomic DNA samples were extracted using the OMEGA Soil DNA Kit（D5625-01）, following the manufacturer's instructions, and stored at -20°C prior to further assessment. The quantity and quality of extracted DNAs were measured using a NanoDrop ND-1000 spectrophotometer (Thermo Fisher Scientific, Waltham, MA,USA) and agarose gel electrophoresis, respectively. The extracted microbial DNA was processed to construct metagenome shotgun sequencing libraries with insert sizes of 400 bp by using Illumina TruSeq Nano DNA LT Library Preparation Kit. Each library was sequenced by Illumina HiSeq X-ten platform (Illumina, USA) with PE150 strategy at Personal Biotechnology Co., Ltd. (Shanghai, China).

Metagenomics Analysis

Raw sequencing reads were processed to obtain quality-filtered reads for further analysis. First, sequencing adapters were removed from sequencing reads using Cutadapt (v1.2.1) (Martin 2011).Secondly, low quality reads were trimmed using a sliding-window algorithm in fastp (Chen, Zhou et al. 2018). Once quality-filtered reads were obtained， taxonomical classifications of metagenomics sequencing reads from each sample were performed using [Kraken2 (Wood, Lu et al. 2019) against an RefSeq-derived database, which included genomes from archaea, bacteria, viruses, fungi, protozoans, metazoans and viridiplantae. Reads assigned to metazoans or viridiplantae were removed for downstream analysis] or [Kaiju (Menzel et al., 2016) with greedy-5 mode against an nr-derived database, which included proteins from archaea, bacteria, viruses, fungi and microbial eukaryotes]. Megahit (v1.1.2)(Liu, Chi-Man et al. 2015) was used to assemble for each sample using the meta-large presetted parameters. The generated contigs (longer than 200bp) were then pooled together and clustered using mmseqs2 (Steinegger and SöDing 2017) with "easy-linclust" mode, setting sequence identity threshold to 0.95 and covered residuse of the shorter contig to 90%. The lowest common ancestor taxonomy of the non-redundant contigs was obtained by aligning them against the NCBI-nt database by mmseqs2 (Steinegger and SöDing 2017) with "taxonomy" mode, and contigs assigned to Viridiplantae or Metazoa were dropped in the following analysis. MetaGeneMark (Zhu, Alexandre et al. 2010) was used to predict the genes in the contigs. CDS sequences of all samples were clustered by mmseqs2 (Steinegger and SöDing 2017) with "easy-cluster" mode, setting protein sequence identity threshold to 0.90 and covered.

Table S1. Primer sequences and PCR conditions used for qPCR amplification in the study and their respective amplification sizes.

Target gene	Primer	**Sequence**	Amplicon size (bp)	Annealing temperature (°C)	Reference
Int1	intI1-F	CCTCCCGCACGATGATC	280	55	10.1016/j.scitotenv.2018.12.088
	intI1-R	TCCACGCATCGTCAGGC			
16S RNA	1369-F	CGGTGAATACGTTCYCGG	143	55	10.1016/j.scitotenv.2018.12.088
	1492-R	GGWTACCTTGTTACGACTT			
Tetracycline	tetA-F	GCTACATCCTGCTTGCCTTC	210	60	10.1006/mcpr.2001.0363
	tetA-R	CATAGATCGCCGTGAAGAGG			
	tetW-F	GAGAGCCTGCTATATGCCAGC	168	60	10.1016/j.scitotenv.2018.12.088

	tetW-R	GGGCGTATCCACAATGTTAAC			
Sulfonamide	sulI-F	CACCGGAAACATCGCTGCA	158	57	10.1016/j.scitotenv.2018.12.088
	sulI-R	AAGTTCCGCCGCAAGGCT			
	sulII-F	CTCCGATGGAGGCCGGTAT	190	60	10.1016/j.scitotenv.2018.12.088
	sulII-R	GGGAATGCCATCTGCCTTGA			
Beta-lactamase	bla_{TEM-1}-F	AATAAACCAGCCAGCCGGAA	209	53.8	10.1021/acs.est.7b01120
	bla_{TEM-1}-R	TTGATCGTTGGGAACCGGAG			

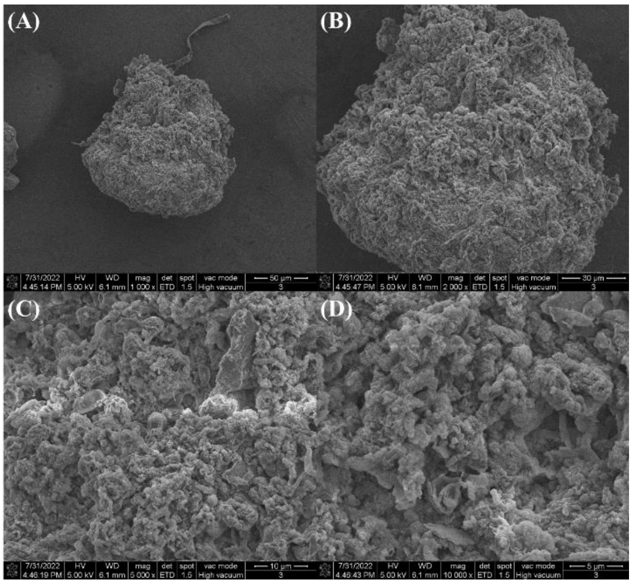

Fig. S1 Microstructure of salt-tolerant aerobic granular sludge by SEM ((A)×1000; (B)×2000; (C)×5000; (D)×10000;)

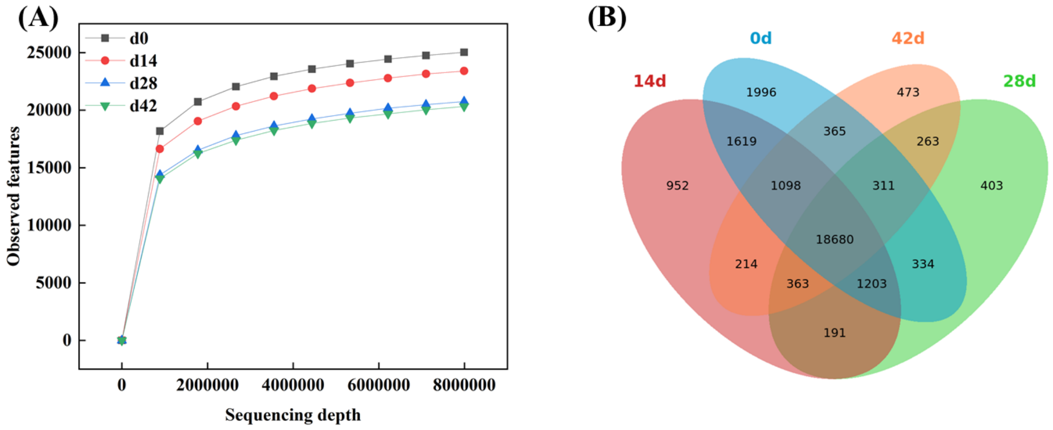

Fig. S2 (A)Rarefaction curve and (B) Venn diagram for sludge samples

Table S2 Annotation of CARD database for SAGS

d0	d14	d28	d42	Antibiotic Resistance Ontology (ARO)	Drug classification	bacterial antimicrobial resistance (AMR)
1.026219	1.290805	0.893908	0	EreA2	macrolide antibiotic	macrolide esterase
5.570515	0	0	0	msrE	macrolide antibiotic;	ABC-F ATP-binding cassette ribosomal protection protein
1.193455	0.750579	0	4.026074	APH(3')-Ia	aminoglycoside antibiotic	APH(3')
0.876824	1.93006	0.286415	0.492989	aadA5	aminoglycoside antibiotic	ANT(3")
0	1.447545	0	0	OXA-1	cephalosporin	OXA beta-lactamase
2.52016	0.109308	0	0	Enterobacter cloacae acrA	fluoroquinolone antibiotic;	resistance-nodulation-cell division (RND) antibiotic efflux pump
0	0.837423	0	0	msrE	macrolide antibiotic;	ABC-F ATP-binding cassette ribosomal protection protein
0.789786	0.596048	0	0.710484	OXA-347	cephalosporin	OXA beta-lactamase
4.584614	6.59045	1.711506	0	sul1	sulfonamide antibiotic	sulfonamide resistant sul
0.2903	0.365147	0	0	OXA-347	cephalosporin	OXA beta-lactamase
0	1.71018	0	0	tet32	tetracycline antibiotic	tetracycline-resistant ribosomal protection protein
1.611164	0	0	0	aadA17	aminoglycoside antibiotic	ANT(3")
0.137004	2.154085	1.074058	0	cmx	phenicol antibiotic	major facilitator superfamily (MFS) antibiotic efflux pump
0.92768	0.697852	0.579932	0	mphE	macrolide antibiotic	macrolide phosphotransferase (MPH)
0	0	1.222505	9.46899	LEN-17	penam	LEN beta-lactamase
0	0.288684	0	0	AAC(3)-IIe	aminoglycoside antibiotic	AAC(3)
1.835259	0.36449	0	0	msrE	macrolide antibiotic;	ABC-F ATP-binding cassette ribosomal protection protein
0.288739	0.726367	1.50907	0	sul4	sulfonamide antibiotic	sulfonamide resistant sul
0.72575	0.182573	0	0	AAC(6')-Ib9	aminoglycoside antibiotic	AAC(6')
0.72575	1.217155	0	0	EreA2	macrolide antibiotic	macrolide esterase

0.873892	0.928842	4.394968	6.356959	tet(A)	tetracycline antibiotic	major facilitator superfamily (MFS) antibiotic efflux pump
0.839148	0.395813	0	0.235903	OXA-21	cephalosporin; penam	OXA beta-lactamase
0.187781	0.708589	0	0	OXA-21	cephalosporin; penam	OXA beta-lactamase
0.706651	0.740703	0	0.529747	Erm(42)	macrolide antibiotic;	Erm 23S ribosomal RNA methyltransferase
2.165543	0	0	0	QnrB12	fluoroquinolone antibiotic	quinolone resistance protein (qnr)
0.40686	1.919094	1.594813	0	aadA3	aminoglycoside antibiotic	ANT(3")
0.801574	0.433026	0	0	catB3	phenicol antibiotic	chloramphenicol acetyltransferase (CAT)
1.094	0.125097	0	0	cmlA5	phenicol antibiotic	major facilitator superfamily (MFS) antibiotic efflux pump
2.314891	1.747037	0	1.041226	mphA	macrolide antibiotic	macrolide phosphotransferase (MPH)
0.375563	0	0	0	CMY-71	cephamycin	CMY beta-lactamase
1.396963	0.390475	0	0	lsaE	macrolide antibiotic;	ABC-F ATP-binding cassette ribosomal protection protein
0.14133	0	0	0	sul1	sulfonamide antibiotic	sulfonamide resistant sul
1.541466	1.523838	0.258936	0	ANT(3")-IIa	aminoglycoside antibiotic	ANT(3")
0.234522	0.294987	0.612854	0	sul4	sulfonamide antibiotic	sulfonamide resistant sul
0.168885	0	0	0	QnrB12	fluoroquinolone antibiotic	quinolone resistance protein (qnr)
